# Identification and analysis of the complete mitochondrial genome of *Thaumetopoea pityocampa* (Lepidoptera: Notodontidae)

**DOI:** 10.1080/23802359.2019.1678422

**Published:** 2019-10-21

**Authors:** Kai Wu, Jinge Yang, Yuyang Ni, Qiuning Liu

**Affiliations:** a College of Life Sciences, Shangrao Normal University, Shangrao, PR China;; b Jiangxi Medical College, Shangrao, PR China;; c Jiangsu Provincial Key Laboratory of Coastal Wetland Bioresources, Jiangsu Synthetic Innovation Center for Coastal Bio-agriculture, Yancheng Teachers University, Yancheng, PR China

**Keywords:** *Thaumetopoea pityocampa*, mitochondrial genome, phylogenetic analysis

## Abstract

The mitochondrial genome (mitogenome) provides important information for phylogenetic analysis and understanding evolutionary origins. *Thaumetopoea pityocampa* is a forest pest that harms nearly all cedar and pine species. In this study, the *T. pityocampa* mitochondrial genome was sequenced, assembled, and annotated. The sequence length of the genome was found to be 15,737 bp, containing 13 protein-coding genes (PCGs), two rRNA genes, 22 tRNA genes, and an A + T-rich region compared with the genomes of other lepidopterans. The overall nucleotide composition is: 37.3% T, 40.5% A, 14.6% C, and 7.6% G, demonstrating an AT bias (A + T: 77.8%). Our phylogenetic tree analysis results showed that *T. pityocampa* and *Ochrogaster lunifer* were the most similar species, with the closest evolutionary distance. The mitogenome sequence determined in this study will contribute to improved understanding of Notodontidae evolution.

Lepidoptera, which contains butterflies and moths, is the second largest order of insects, surpassed only by Coleoptera. Noctuoidea is among the largest superfamilies within Lepidoptera, with almost 42,400 described species*. Thaumetopoea pityocampa* (Lepidoptera: Notodontidae) is a pest that consumes the needles of conifers such as *Cedrus*, *Pinus,* and *Pseudotsuga* (Colacci et al. 2018). Besides damaging forest production, the setae of *T. pityocampa* are also a threat to human and animal health because they contain allergens such as Tha p 1 and Tha p 2 (Rebollo et al. 2002; Vega et al. 2011; Rodriguez-Mahillo et al. 2012; Kaszak et al. 2015; Berardi et al. 2017). Insect genomic information improves our understanding of various aspects of pests such as their physiology, biochemistry, reproduction, migration, and tolerance to extreme environments. The *T. pityocampa* genome is 537 Mb in length; *de novo* transcriptomic analysis of two phenologically divergent populations has identified 9625 unigenes while 29,701 *bona fide* unigenes found in samples from different developmental stages (Gschloessl et al. 2014; Gschloessl et al. 2018). Although mitochondrial gene fragments of *T. pityocampa* have been sequenced and compared with those from other species or different locations, complete mitochondrial genome sequencing remains to be performed and is necessary for phylogenetic and evolutionary research (Kerdelhue et al. 2009; Rousselet et al. 2010). In this study, we report the complete mitochondrial genome of *T. pityocampa*.

*T. pityocampa* eggs used for studying were collected in October 2017 in Venosta, Italy (46°37′N, 10°46′E) and extracted DNA (YTU-20171001008) was stored at Jiangsu Provincial Key Laboratory of Coastal Wetland Bioresources in Yancheng Teachers University. The National Centre for Biotechnology Information (NCBI) BLAST (http://blast.ncbi.nlm.nih.gov/Blast) and DNAStar packages (DNAStar Inc. Madison, WI) were used to annotate the genome sequence. The MAFFT sequence alignment programme was used to compare genome sequences from *T. pityocampa* and other species (Katoh et al. 2002). Bayesian inference (BI) and maximum likelihood (ML) analyses were performed using the MrBayes version 3.2.1 and IQ-TREE software, respectively. We selected mtMet + F + I + G4 as the best-fit model for amino acid sequences, as determined by the Modelfinder tool using the Bayesian information criterion (BIC). The mitochondrial genome (mitogenome) is considered a powerful marker for resolving phylogenetic relationships (Galtier et al. 2009). To analyse phylogenetic relationships, we obtained the complete mitogenomes of other species from the GenBank database, and aligned the amino acid sequences of the 13 PCGs using ML and BI methods to reconstruct the phylogenetic tree.

Like those of other lepidopterans, the *T. pityocampa* mitochondrial genome (GenBank accession no. MH286070) has 37 functional genes and a long-chain non-coding region (AT enrichment region), at a length of 15,737 bp. The overall nucleotide composition is: 37.3% T, 40.5% A, 14.6% C, and 7.6% G, demonstrating an AT bias (A + T: 77.8%).

From the BI tree of concatenated amino acid sequences from the 13 PCGs, phylogenetic analysis showed that *T. pityocampa* is very closely related to *Ochrogaster lunifer* (Figure 1). *Thaumetopoea pityocampa* was phylogenetically distant from two the outgroup species, *Phthonandria atrilineata* and *Biston panterinaria*.

**Figure 1.**
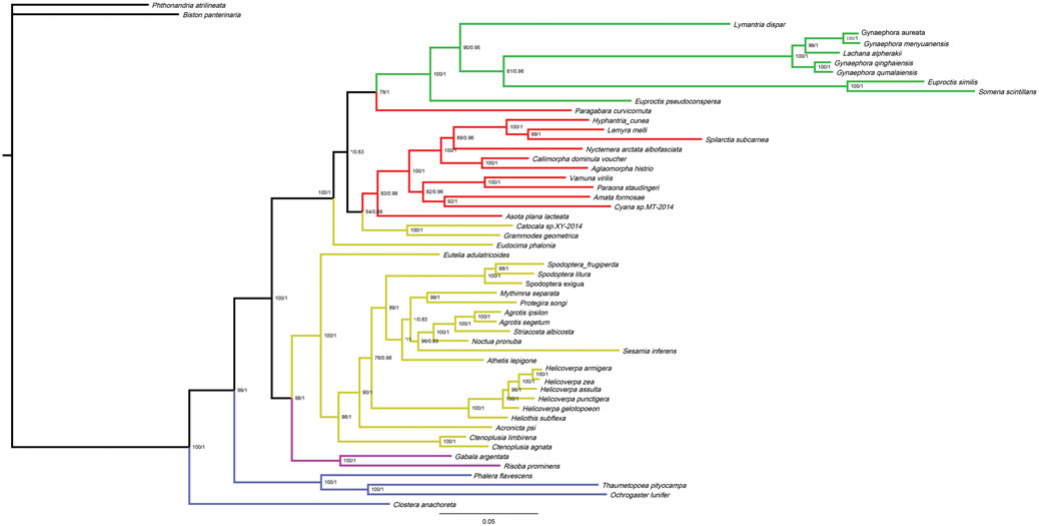
Phylogenetic tree of *Thaumetopoea pityocampa* and 52 other insect species, constructed based on 13 protein-coding genes. *Phthonandria atrilineata* (GenBank accession no. EU569764) and *Biston panterinaria* (JX406146) were included as outgroups. GeneBank accession numbers of other species: *Lymantria dispar* (FJ617240), *Gynaephora aureata* (KJ507132), *Gynaephora menyuanensis* (KC185412), *Lachana alpherakii* (KJ957168), *Gynaephora qinghaiensis* (KJ507133), *Gynaephora qumalaiensis* (KJ507134), *Euproctis similis* (KT258910), *Somena scintillans* (MH051839), *Euproctis pseudoconspersa* (KJ716847), *Paragabara curvicornuta* (KT362742), *Hyphantria cunea* (GU592049), *Lemyra melli* (KP307017), *Spilarctia subcarnea* (KT258909), *Nyctemera arctata albofasciata* (KM244681), *Callimorpha dominula voucher* (KP973953), *Aglaomorpha histrio* (KY800518), *Vamuna virilis* (KJ364659), *Paraona staudingeri* (KY827330), *Amata formosae* (KC513737), *Cyana sp. MT-2014* (KM244679), *Asota plana lacteata* (KJ173908), *Catocala sp. XY-2014* (KJ432280), *Grammodes geometrica* (KY888135), *Eudocima phalonia* (KY196412), *Eutelia adulatricoides* (KJ185131), *Spodoptera frugiperda* (KM362176), *Spodoptera litura* (JQ647918), *Spodoptera exigua* (JX316220), *Mythimna separata* (KM099034), *Protegira songi* (KY379907), *Agrotis ipsilon* (KF163965), *Agrotis segetum* (KC894725), *Striacosta albicosta* (KM488268), *Noctua pronuba* (KJ508057), *Sesamia inferens* (JN039362), *Athetis lepigone* (MF152835), *Helicoverpa armigera* (GU188273), *Helicoverpa zea* (KJ930516), *Helicoverpa assulta* (KR149448), *Helicoverpa punctigera* (KF977797), *Helicoverpa gelotopoeon* (MG437199), *Heliothis subflexa* (KT598688), *Acronicta psi* (KJ508060), *Ctenoplusia limbirena* (KM244665), *Ctenoplusia agnata* (KC414791), *Gabala argentata* (KJ410747), *Risoba prominens* (KJ396197), *Phalera flavescens* (JF440342), *Ochrogaster lunifer* (AM946601), and *Clostera anachoreta* (KX108766). Numbers above branches indicate bootstrap support values for phylogenetic trees produced using maximum likelihood and Bayesian inference analyses.
